# Has the quality of reporting improved since it became mandatory to use the Standards for Reporting Diagnostic Accuracy?

**DOI:** 10.1186/s13244-023-01432-7

**Published:** 2023-05-15

**Authors:** Ann-Christine Stahl, Anne-Sophie Tietz, Marc Dewey, Benjamin Kendziora

**Affiliations:** 1 Department of Radiology, Charité – Universitätsmedizin Berlin (Corporate Member of Freie Universität Berlin and Humboldt-Universität zu Berlin), Berlin, Germany; 2 Department of Dermatology and Allergy, University Hospital, Ludwig Maximilian University, Munich, Germany

**Keywords:** Checklist, Accuracy, Diagnostic tests, Reference standards, Research design

## Abstract

**Objectives:**

To investigate whether making the Standards for Reporting Diagnostic Accuracy (STARD) mandatory by the leading journal ‘Radiology’ in 2016 improved the quality of reporting of diagnostic accuracy studies.

**Methods:**

A validated search term was used to identify diagnostic accuracy studies published in Radiology in 2015 and 2019. STARD adherence was assessed by two independent reviewers. Each item was scored as yes (1 point) if adequately reported or as no (0 points) if not. The total STARD score per article was calculated. Wilcoxon–Mann–Whitney tests were used to evaluate differences of the total STARD scores between 2015 and 2019. In addition, the total STARD score was compared between studies stratified by study design, citation rate, and data collection.

**Results:**

The median number of reported STARD items for the total of 66 diagnostic accuracy studies from 2015 to 2019 was 18.5 (interquartile range [IQR] 17.5–20.0) of 29. Adherence to the STARD checklist significantly improved the STARD score from a median of 18.0 (IQR 15.5–19.5) in 2015 to a median of 19.5 (IQR 18.5–21.5) in 2019 (*p* < 0.001). No significant differences were found between studies stratified by mode of data collection (prospective vs. retrospective studies, *p* = 0.68), study design (cohort vs. case–control studies, *p* = 0.81), and citation rate (two groups divided by median split [< 0.56 citations/month vs. ≥ 0.56 citations/month], *p* = 0.54).

**Conclusions:**

Making use of the STARD checklist mandatory significantly increased the adherence with reporting standards for diagnostic accuracy studies and should be considered by editors and publishers for widespread implementation.

**Critical relevance statement:**

Editors may consider making reporting guidelines mandatory to improve the scientific quality.

**Graphical Abstract:**

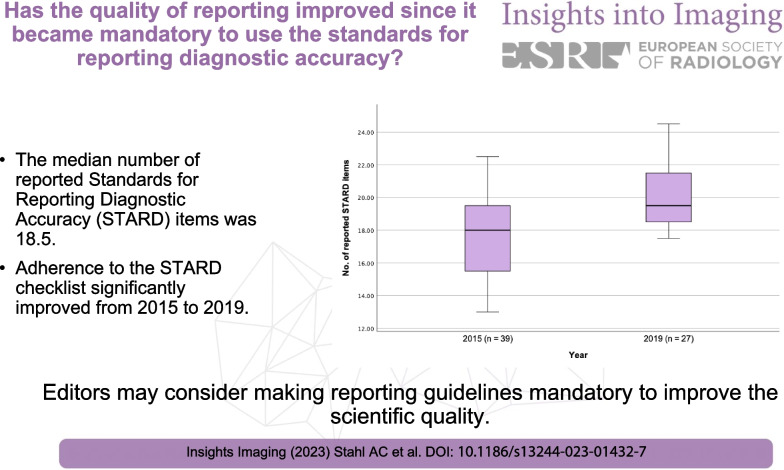

**Supplementary Information:**

The online version contains supplementary material available at 10.1186/s13244-023-01432-7.

## Key points

- The median number of reported STARD items for all studies was 18.5.
- Adherence to the STARD checklist significantly improved from 2015 to 2019.
- Editors may consider making reporting guidelines mandatory to improve the scientific quality.

**Supplementary Information:**

The online version contains supplementary material available at 10.1186/s13244-023-01432-7.

## Introduction

Diagnostic accuracy studies play an important role in introducing a new diagnostic test into clinical practice [1] because diagnostic test accuracy compared with an established reference standard provides information about how well diagnostic tests may improve clinical decision making [2]. Diagnostic accuracy studies are at risk of bias [3, 4] because measures of diagnostic accuracy, such as sensitivity and specificity, are not fixed values but reflect the performance of the index test under certain study and test circumstances [2, 4–6]. Therefore, a detailed description of the methodology, setting, and subjects is crucial for readers to judge the trustworthiness of the results (internal validity) and appraise the applicability of the medical test in clinical practice (external validity, i.e., generalizability) [5].

In the past, studies published in journals with high impact factors had shortcomings in reporting diagnostic accuracy, leading to overestimation of test performance and improper recommendations with disadvantages for patient outcomes [7]. Furthermore, “incomplete reporting has been identified as a major source of avoidable waste in biomedical research” [8] and growing health care costs [9, 10]. Following the successful CONSORT (Consolidated Standards of Reporting Trials) initiative [11], the Standards for Reporting Diagnostic Accuracy (STARD) statement was published in 2003 [12] and updated in 2015 [8]. It consists of a checklist of 30 essential items to guide authors in planning and reporting diagnostic accuracy studies [8]. Since then, STARD has been endorsed by more than 200 biomedical journals [13].

In February 2016, the use of reporting guideline checklists became mandatory for all original research manuscripts submitted to Radiology, which had endorsed STARD since its publication [14, 15]. We used this as an opportunity to investigate the reporting quality of diagnostic accuracy studies published in Radiology before and after guideline implementation and to evaluate whether reporting quality improved after mandating reporting guideline use. Further, we analyzed whether the total STARD score differed between studies stratified by study design, citation rate, and data collection.

## Methods

This analysis, even not fulfilling all criteria of a meta-analysis, complied with the Preferred Reporting Items for Systematic Reviews and Meta-Analyses (PRISMA) guidelines [16]. Our analysis was therefore not eligible for registration in the international prospective register of systematic reviews (PROSPERO) [17].

### Literature search

To identify diagnostic accuracy studies published in Radiology in 2015 and 2019, we performed a systematic literature review in MEDLINE (using PubMed) using a validated search strategy proposed by Devillé et al. [18], which served as the basis (Additional file 1: Table S1) for our search strategy. The search strategy is detailed in Additional file 1: Table S2. Additionally, we manually searched the website of Radiology for additionally eligible studies which were not identified in MEDLINE. PubMed was last searched on April 8, 2020; the website of Radiology on June 23, 2020.

### Study selection

Articles were included if (1) there was at least one measure of diagnostic accuracy (sensitivity, specificity, likelihood ratios, predictive values, area under the receiver operator curve, accuracy), (2) the results of at least one medical imaging test were compared against a reference standard, and (3) the study was conducted in human subjects. Articles dealing with predictive or prognostic accuracy as well as commentaries, editorials, letters, reviews and the development of models were excluded. Two reviewers (A.S., an advanced medical student, with 3 years of experience in performing literature reviews of diagnostic accuracy studies, and A.T., a dentist, with 1 year of experience in this field) independently reviewed all studies for inclusion; discrepancies were resolved in consensus meetings with a third reviewer (B.K., a physician with 8 years of experience in radiological research). First, we went through all titles, keywords, and abstracts to identify potentially eligible articles. Finally, the full texts of the articles remaining after this step were assessed for eligibility. The following information was extracted from each included article: publication date (2015 vs. 2019), mode of data collection (prospective vs. retrospective), and study design (cohort vs. case–control study).

### Adherence to STARD

Although two studies reported good reproducibility of the STARD checklist [19, 20], two reviewers (A.S., A.T.) independently pilot-tested the STARD checklist on four articles from 2014 TO 2020. Uncertainties regarding the explanation and elaboration of each item were discussed to make sure that the reviewers agreed about the interpretation of the STARD criteria. For the purpose of our analysis, we excluded item 11 (rationale for choosing the reference standard (if alternatives exist)) from the STARD checklist following the approach of Wilczynski [5, 21] because in case that no information regarding this item was found in an article, it was not possible to reliably determine whether the authors simply forgot to mention it in the manuscript or ignored it in their study because no alternatives existed. Thus, the finally used checklist consisted of 29 items. Each adequately reported item was scored yes (1 point) or no (0 points). As items 10, 12, 13, and 21 refer to both the index test and the reference standard, we split these items and counted each of the two modalities as ½ item (0.5 points). Both reviewers (A.S., A.T.) evaluated independently all included articles according to the 29-item checklist. Discrepancies were resolved in consensus meetings. If no consensus could be reached, a third reviewer (B.K.) helped to make the final decision. Reviewers were not blinded to journal, publication year, and authors. The reviewers did not evaluate the methodological quality [22] of the study but the quality of reporting [8].

### Data and statistical analysis

We calculated the total STARD score for each included article by adding the number of reported STARD items (range, 0–29). The median and interquartile range (IQR) for the total STARD scores were calculated. Assuming that each item is of equal weight, a higher score suggests a better reporting quality. Wilcoxon–Mann–Whitney’s test was used to compare the STARD score between papers published in 2015 and papers published in 2019. This comparison was performed with inclusion of all studies as well as with inclusion of the following subgroups: prospective studies, retrospective studies, cohort studies, case control studies, studies with a citation rate above median, and studies with citation rate below median. In addition, Wilcoxon–Mann–Whitney’s test was applied to analyze whether the total STARD score differed between studies stratified by study design (cohort vs. case control studies), citation rate (equal or above vs. below median citation rate), and data collection (prospective vs. retrospective). Vargha and Delaney’s A was used as effect size measure.

The citation rate was calculated by dividing the total number of times each article had been cited by April 30, 2021, by the total number of months since publication (print version). These numbers were provided by the citation index reported in Web of Science (Thomson Reuters, New York, NY, USA).

Cohen´s κ statistics was used to calculate interrater reliability. According to Landis and Koch [23], a *κ* value of 0.4–0.60 indicates moderate; a *κ* value of 0.61–0.80, substantial; and a *κ* value of 0.81–1.00, (almost) perfect agreement between the reviewers.* p* values less than < 0.05 were considered statistically significant. The code for the statistical analysis was written in R language, version 4.2.0.

## Results

### Search results and study characteristics

**Fig. 1:**
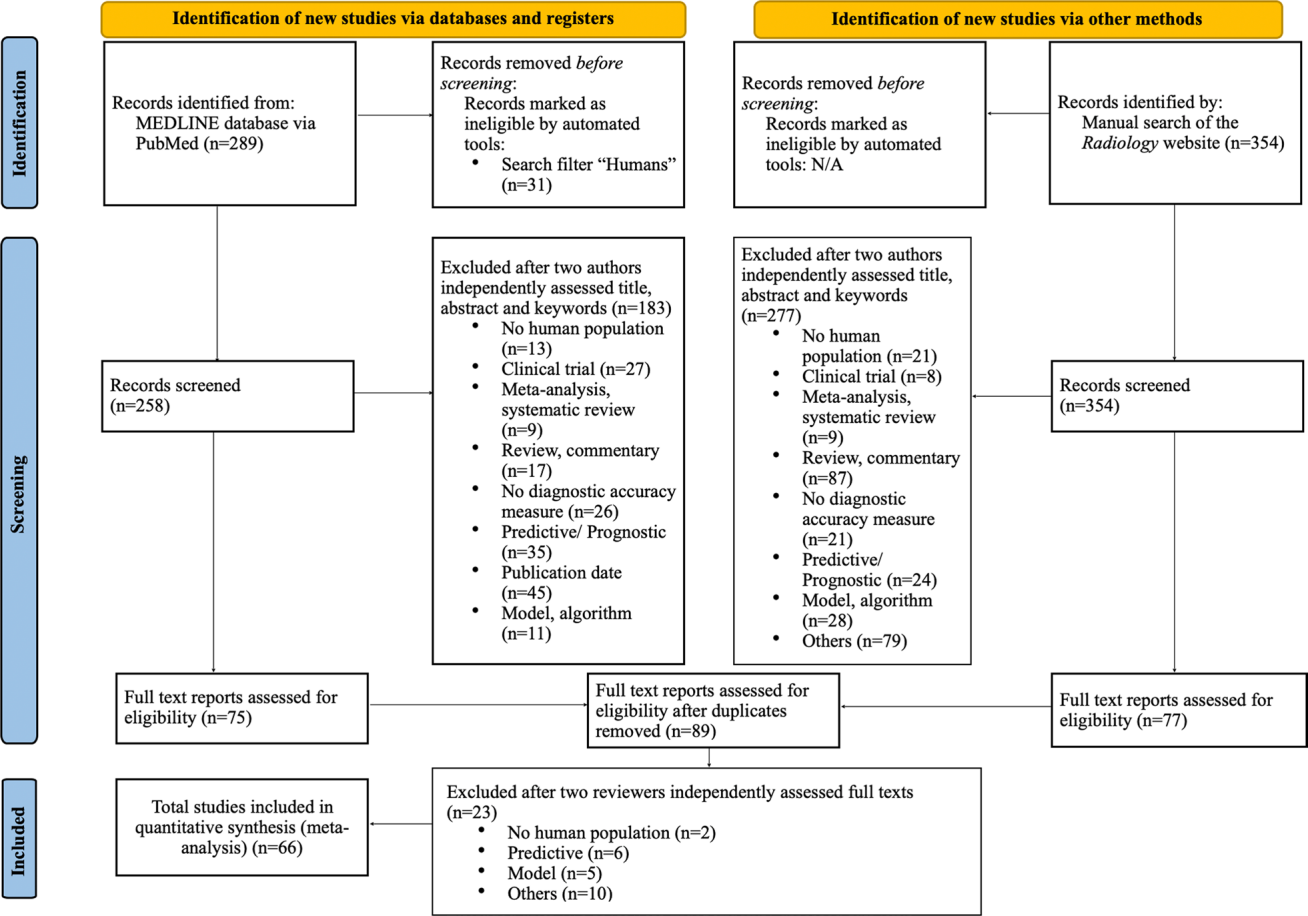
PRISMA (Preferred Reporting Items for Systematic Reviews and Meta-Analyses) 2020 flow diagram of selected diagnostic accuracy studies published in Radiology in 2015 and 2019 with detailed description for exclusion criteria. N/A, not applicable

The systematic literature search identified 289 publications, the manual search 354. Independent assessment of title, abstract, and keywords according to our inclusion criteria by two reviewers (A.S., A.T.) identified 75 potentially relevant articles from the literature search and 77 from the manual search. After exclusion of 63 duplicates, two readers (A.S., A.T.) independently examined the full texts of 89 articles. The multilevel selection process finally led to the identification of 66 eligible articles. The PRISMA 2020 flow diagram (Fig. 1) [16] provides detailed information on the study selection process. The median citation rate was 0.56 citations per month (range: 0.1–2.35). Baseline characteristics of all studies included are compiled in Table 1.

**Table 1:** Characteristics of included studies published in Radiology

Characteristic	All Articles (*n* = 66), *n* (%)	2015 (*n* = 39), *n* (%)	2019 (*n* = 27), *n* (%)
Publication year ^a^		39 (59)	27 (41)
*Mode of data collection*			
Retrospective	32 (48)	16 (41)	16 (59)
Prospective	34 (52)	23 (59)	11 (41)
*Study design*			
Case control	9 (14)	5 (13)	4 (15)
Cohort	57 (86)	34 (87)	23 (85)
*Citation rate (median split)*^b^			
Infrequently	33 (50)	21 (54)	12 (44)
Frequently	33 (50)	18 (46)	15 (56)

Unless otherwise indicated, data are numbers of studies and data in parentheses are percentages

^a^2015: STARD (Standards for Reporting Diagnostic Accuracy) recommended; 2019: STARD mandatory

^b^Infrequently < 0.56 citations/month; Frequently ≥ 0.56 citations/month

### Adherence to STARD

The median number of reported STARD items for the 66 diagnostic accuracy studies analyzed was 18.5 (IQR 17.5–20.0) of 29, with a range of 13 to 24.5. A list of all included studies with individual total STARD scores is provided in Additional file 1: Table S3.

Overall agreement in scoring the 33 (sub-)items was 85% (1859/2178). Based on a Cohen´s *κ* statistic of 0.70 (95% CI 0.66, 0.73), interrater reliability was substantial.

Studies published in 2019 showed a 2.2 points higher (95% CI 1.2–3.3, *p* < 0.001, Vargha and Delaney’s *A* = 0.24) total STARD score than those published in 2015 (19.5 [IQR 18.5–21.5] vs. 18.0 [15.5–19.5]), indicating a better overall reporting quality. A significant difference in the median STARD score between 2015 and 2019 was also found for subgroups consisting of prospective studies (3.3, 95% CI 1.6–5.0, p < 0.001, Vargha and Delaney’s *A* = 0.14), cohort studies (2.0, 95% CI 0.8–3.1, *p* = 0.002, Vargha and Delaney’s *A* = 0.28), case control studies (4.15, 95% CI 1.0–7.3, *p* = 0.017, Vargha and Delaney’s *A* = 0.0), studies with a citation rate above median (2.5, 95% CI 0.9–4.1, *p* = 0.003, Vargha and Delaney’s *A* = 0.23), and studies with a citation rate below median (2.2, 95% CI 0.6–3.8, *p* = 0.008, Vargha and Delaney’s *A* = 0.24). Retrospective studies did not show a significant difference in the reporting quality between 2015 and 2019 (1.4, 95% CI − 0.1 to 2.9, *p* = 0.065, Vargha and Delaney’s *A* = 0.36).

No difference in the total STARD score was found between studies stratified by mode of data collection (*p* = 0.68, Vargha and Delaney’s *A* = 0.47), study design (*p* = 0.81, Vargha and Delaney’s *A* = 0.53), and citation rate (*p* = 0.54, Vargha and Delaney’s *A* = 0.54). Detailed results are provided in Table 2.

**Table 2:** Summary of Wilcoxon–Mann–Whitney tests

Categorical variable	Summary of finding
Publication year	Studies published in 2019 (STARD mandatory) reported on more items compared to those in 2015 (STARD recommended) (19.5 [IQR 18.5–21.5] vs. 18.0 [IQR 15.5–19.5], *p* < 0.001, Vargha and Delaney’s *A* = 0.24)
Mode of data collection	No evidence of a difference in the total STARD score was found for data collection (retrospective, 18.8 [IQR 17.9–19.6] vs. prospective, 18.5 [IQR 16.8–20.0], *p* = 0.68, Vargha and Delaney’s *A* = 0.47)
Study design	No evidence of a difference in the total STARD score was found for study design (case control, 18.5 [IQR 17.5–19.0] vs. cohort, 18.5 [IQR 17.5–20.0], *p* = 0.81, Vargha and Delaney’s *A* = 0.53)
Citation rate (median split)^a^	No evidence of a difference in the total STARD score was found for citation rate (infrequently, 18.5 [IQR 18.0–20.5] vs. frequently, 18.5 [IQR 17.5–19.5], *p* = 0.54, Vargha and Delaney’s *A* = 0.54)

*STARD* Standards for Reporting Diagnostic Accuracy

^a^Infrequently < 0.56 citations/month; Frequently ≥ 0.56 citations/month

### Item-specific adherence to STARD

**Fig. 2:**
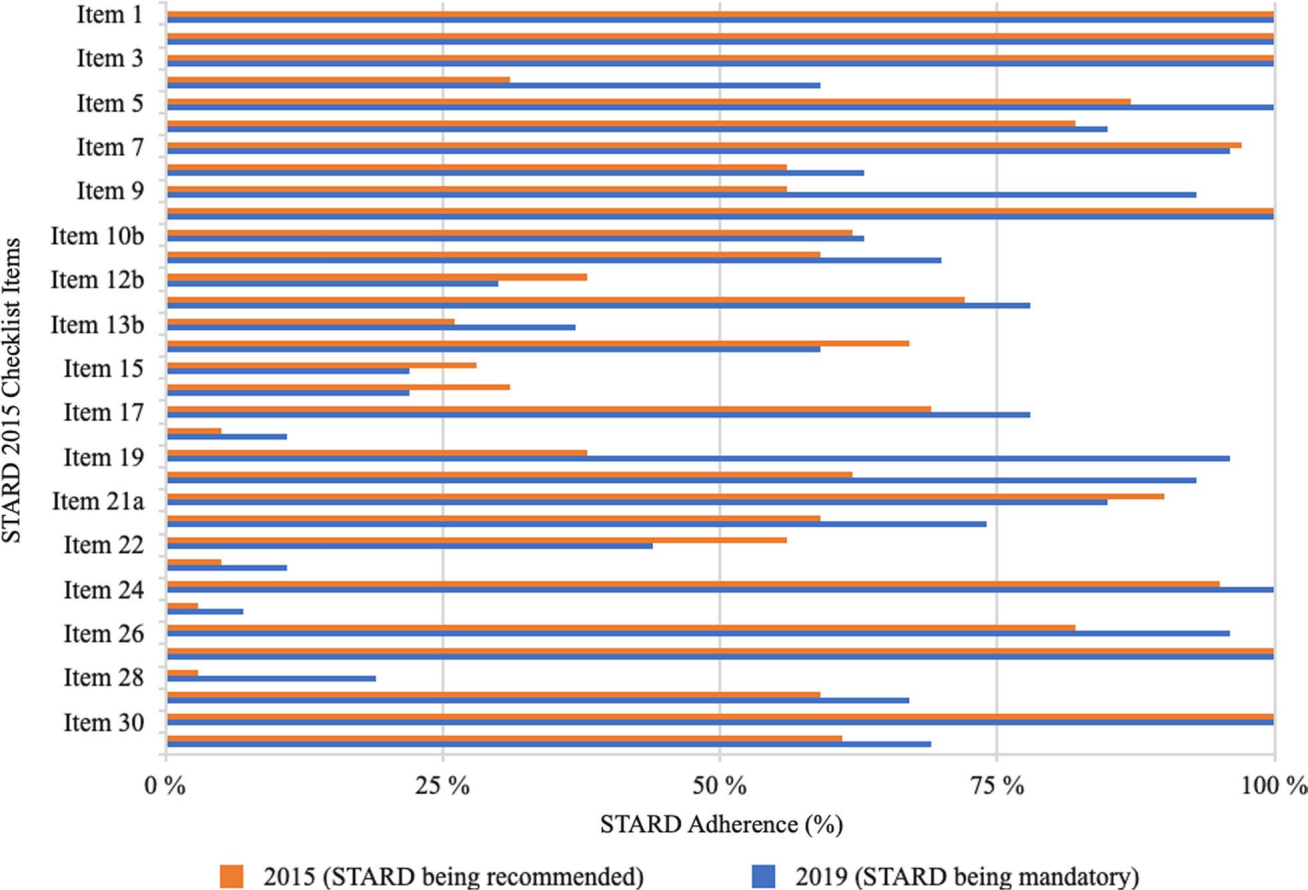
Graph shows results of comparison of overall adherence (%) to 29 STARD 2015 checklist items and reporting of individual items between studies published in 2015, with STARD being recommended, and those published in 2019, with STARD being mandatory. Studies published in 2019 adhered in general to more items of the STARD 2015 checklist. However, items 12b (prespecified definition of test positivity cutoffs of the reference standard), 14 (methods for estimating diagnostic accuracy measures), 15 and 16 (handling of missing and indeterminate results) and item 22 (time interval between tests) were more often reported in 2015. When referring to the split items 10, 12, 13 and 21, the graph reveals that information about the index test (a items) was more frequently reported than for the reference standard (b items). STARD, Standards for Reporting Diagnostic Accuracy

The results for adherence to individual STARD items and comparisons of reporting frequencies between studies published in 2015 and 2019 are shown in Table 3 and Fig. 2. Although STARD has been mandatory in Radiology since 2016, seven relevant items of the STARD checklist (item 12b (prespecified definition of test positivity cutoffs of the reference standard), items 15 and 16 (handling of missing and indeterminate results), 18 (sample size calculation), 23 (a cross-tabulation), 25 (adverse events), and 28 (registration number) were infrequently reported (< 33%) in the 27 diagnostic accuracy studies published in Radiology in 2019 with very poor reporting rates for items 18 and 23 (11%, 3/27). Providing a registration number, however, notably improved by 16%-points between 2015 (3%, 1/39) and 2019 (19%, 5/27). Six of the 33 (sub-)items were moderately (33–66% of studies) reported in 2019. Especially reporting of study objectives and hypotheses (item 4) nearly doubled between 2015 (31%, 12/39) and 2019 (59%, 16/27). Last, 20 of 33 items were frequently (> 66%) reported by studies published in 2019. Particularly item 9 (sample selection) improved by 37%-points (56%, 22/39 vs. 93%, 25/27), item 19 (flow diagram) by 58%-points (38%, 15/39 vs. 96%, 26/27), and item 20 (baseline demographics) by 31%-points (62%, 24/39 vs. 93%, 25/27) between 2015 and 2019. For all items (items 10, 12, 13, 21) concerning both the index test and reference standard, the information was more frequently reported for the index test.

**Table 3:** Reporting frequencies of individual STARD items for all studies and comparison of reporting frequencies with STARD being recommended (2015) vs. STARD being mandatory (2019) in Radiology

STARD item No.	Item description	All Articles (*n* = 66), %	Articles published in 2015 (*n* = 39), %	Articles published in 2019 (*n* = 27), %
*Title or abstract*				
1	Identification as a study of diagnostic accuracy using at least one measure of accuracy (such as sensitivity, specificity, predictive values or AUC)	100 (*n* = 66)	100 (*n* = 39)	100 (*n* = *27*)
2*	Structured summary of study design, methods, results and conclusions (for specific guidance, see STARD for Abstracts)	100 (*n* = 66)	100 (*n* = 39)	100 (*n* = 27)
*Introduction*				
3*	Scientific and clinical background, including the intended use and clinical role of the index test	100 (*n* = 66)	100 (*n* = 39)	100 (*n* = 27)
4*	Study objectives and hypotheses	42 (*n* = 28)	31 (*n* = 12)	59 (*n* = 16)
*Methods*				
5	Whether data collection was planned before the index test and reference standard were performed (prospective study) or after (retrospective study)	92 (*n* = 61)	87 (*n* = 34)	100 (*n* = 27)
6	Eligibility criteria	83 (*n* = 55)	82 (*n* = 32)	85 (*n* = 23)
7	On what basis potentially eligible participants were identified (such as symptoms, results from previous tests, and inclusion in registry)	97 (*n* = 64)	97 (*n* = 38)	96 (*n* = 26)
8	Where and when potentially eligible participants were identified (setting, location and dates)	59 (*n* = 39)	56 (*n* = 22)	63 (*n* = 17)
9	Whether participants formed a consecutive, random or convenience series	71 (*n* = 47)	56 (*n* = 22)	93 (*n* = 25)
10a	Index test, in sufficient detail to allow replication	100 (*n* = 66)	100 (*n* = 39)	100 (*n* = 27)
10b	Reference standard, in sufficient detail to allow replication	62 (*n* = 41)	62 (*n* = 24)	63 (*n* = 17)
12a	Definition of and rationale for test positivity cutoffs or result categories of the index test, distinguishing prespecified from exploratory	64 (*n* = 42)	59 (*n* = 23)	70 (*n* = 19)
12b	Definition of and rationale for test positivity cutoffs or result categories of the reference standard, distinguishing prespecified from exploratory	35 (*n* = 23)	38 (*n* = 15)	30 (*n* = 8)
13a	Whether clinical information and reference standard results were available to the performers or readers of the index test	74 (*n* = 49)	72 (*n* = 28)	78 (*n* = 21)
13b	Whether clinical information and index test results were available to the assessors of the reference standard	30 (*n* = 20)	26 (*n* = 10)	37 (*n* = 10)
14	Methods for estimating or comparing measures of diagnostic accuracy	64 (*n* = 42)	67 (*n* = 26)	59 (*n* = 16)
15	How indeterminate index test or reference standard results were handled	26 (*n* = 17)	28 (*n* = 11)	22 (*n* = 6)
16	How missing data on the index test and reference standard were handled	27 (*n* = 18)	31 (*n* = 12)	22 (*n* = 6)
17	Any analyses of variability in diagnostic accuracy, distinguishing prespecified from exploratory	73 (*n* = 48)	69 (*n* = 27)	78 (*n* = 21)
18*	Intended sample size and how it was determined	8 (*n* = 5)	5 (*n* = 2)	11 (*n* = 3)
*Results*				
19	Flow of participants, using a diagram	62 (*n* = 41)	38 (*n* = 15)	96 (*n* = 26)
20	Baseline demographic and clinical characteristics of participants	74 (*n* = 49)	62 (*n* = 24)	93 (*n* = 25)
21a	Distribution of severity of disease in those with the target condition	88 (*n* = 58)	90 (*n* = 35)	85 (*n* = 23)
21b	Distribution of alternative diagnoses in those without the target condition	65 (*n* = 43)	59 (*n* = 23)	74 (*n* = 20)
22	Time interval and any clinical interventions between index test and reference standard	52 (*n* = 34)	56 (*n* = 22)	44 (*n* = 12)
23	Cross-tabulation of the index test results (or their distribution) by the results of the reference standard	8 (*n* = 5)	5 (*n* = 2)	11 (*n* = 3)
24	Estimates of diagnostic accuracy and their precision (such as 95% CIs)	97 (*n* = 64)	95 (*n* = 37)	100 (*n* = 27)
25	Any adverse events from performing the index test or the reference standard	5 (*n* = 3)	3 (*n* = 1)	7 (*n* = 2)
*Discussion*				
26*	Study limitations, including sources of potential bias, statistical uncertainty and generalizability	88 (*n* = 58)	82 (*n* = 32)	96 (*n* = 26)
27*	Implications for practice, including the intended use and clinical role of the index test	100 (*n* = 66)	100 (*n* = 39)	100 (*n* = 27)
*Other information*				
28*	Registration number and name of registry	9 (*n* = 6)	3 (*n* = 1)	19 (*n* = 5)
29*	Where the full study protocol can be accessed	62 (*n* = 41)	59 (*n* = 23)	67 (*n* = 18)
30*	Sources of funding and other support; role of funders	100 (*n* = 66)	100 (*n* = 39)	100 (*n* = 27)

*STARD* Standards for Reporting Diagnostic Accuracy; *Item-No.* item number; *AUC* area under the curve

*Indicates new STARD 2015 items

## Discussion

Shortcomings in reporting diagnostic accuracy studies hamper an objective assessment of the clinical performance of diagnostic tests [24]. To improve reporting quality, the STARD statement was developed [12]. In our analysis, we assessed the reporting quality of 66 diagnostic accuracy studies published before and after using the STARD guidelines became mandatory. We found that (1) adherence to the STARD 2015 checklist was moderate (median 18.5 of 29 items), (2) mandating guideline use had a significant effect on the total STARD score (*p* < 0.001), and (3) that further improvement is especially necessary to ensure adequate reporting of items that are prone to bias and variation [3, 8], such as prespecified definitions of test positivity cutoffs, handling of indeterminate and missing results, providing sample size calculations, and cross-tabulations.

Compared with a previous study by this author group, we found a higher average number of reported items than in diagnostic accuracy studies published in European Radiology [25]. This could be due to the fact that European Radiology is a STARD-endorsing journal, while the use of the STARD checklist is mandatory for studies submitted to Radiology. Therefore making STARD and other checklists mandatory may be considered by Insights into Imaging and other journals of the European Society of Radiology Journal Family to improve the scientific quality. Nevertheless, we found no significant differences in the STARD score between studies stratified by study design, data collection, and citation rate within the studies published in European Radiology as well. This is in-line with the results of this assessment. Choi et al. analyzed 63 studies published from 2011 to 2015 in the Korean Journal of Radiology, a STARD-endorsing journal [26]. The mean total STARD score of their analysis was 20 of 27 items (74%), indicating a relatively high overall reporting quality. This could be due to the fact that the authors excluded item 28 (providing a registration number). In our study, we found the lowest adherence rate for this item (9%, 6/66), which might have affected our total scores. Furthermore, Choi et al. also found no effect of the citation rate on STARD adherence. This is in-line with the results reported by Hogan et al. [27] in 2020 and in contrast with the results of the large assessment by Dilauro et al. [28] who found a weak positive correlation between the total STARD score and the citation rate. Most of the above-mentioned studies additionally compared the reporting quality of diagnostic accuracy studies in journals that had endorsed STARD with those that did not. Their results revealed that STARD endorsement had a relevant impact on the total STARD score [26, 27, 29]. To the best of our knowledge, ours is the first investigation explicitly assessing the impact of mandatory guideline use on reporting quality over time.

A summary of the relevant literature on STARD adherence is provided in Table 4.

**Table 4:** Summary of results of relevant studies dealing with the reporting quality in diagnostic accuracy studies using the STARD Checklist

First author	Study year	Article year	Medical field	Included studies	Mean total STARD Score ^a^	Important findings
Hong et al.^*^	2018	2016	Imaging/Magnetic Resonance	142	16.6/30 (55)	Articles published in journals with higher IFs (17.2 vs. 16; *p* = 0.001) and STARD-adopting journals (17.5 vs. 16.4; *p* = 0.01) achieved higher total STARD scores. No evidence of a difference in the total STARD score was found for mode of data collection and imaging modality
Zarei et al.^*, b^	2018	2015	Radiology	151	(69.45)	Several items, such as providing a registration number (1.1%), full study protocol (10.7%), reporting adverse events (14.9%), a prespecified sample size (16.11%), analyses prespecified from exploratory (28.19%), and the distribution of alternative diagnoses (26.17%) were infrequently reported
Choi et al.^*^	2016	2011–2015	Radiology	63	20/27 (74)	With the effect of exposure time partialled out, the STARD score did not significantly correlate with citation numbers (partial correlation coefficient = 0.15, *p* = 0.23)
Hogan et al.^*^	2020	2018	Pathology	171	15.4/34 (45)	Articles that were published in STARD-adopting journals (16.1 vs. 14.8, *p* = 0.018) reported significantly more items compared to STARD-nonadopting journals. No evidence of a difference in the total STARD score was found for IF, citation number, and pathology (sub-)specialty
Michelessi et al.^*^	2017	2003–2014	Ophthalmology/glaucoma	106	16.8/31 (54.1)	An increase in the total STARD score was found for publication year (OR: 1.03 per year, *p* = 0.03) and for journals with IF > 3.5 vs. < 2 (OR: 1.22, *p* = 0.03)
Korevaar et al	2014	2012	General Medicine	112	15.3/25 (61)	Articles published in 2012 reported on 1.7 items (95% Cl 0.9–2.5) more than in 2004 ^c^. Significantly more items were reported in studies published in general journals than in discipline-specific journals (17.7 vs 14.8, *p* = 0.002), for single gate studies vs. multiple gate studies (16.8 vs. 12.1, *p* < 0.001), and for studies assessing imaging tests compared with laboratory tests and other types of tests (17 vs. 14 vs. 14.5; *p* < 0.001)
Walther et al	2014	2003–2011	Imaging/CT angiography	130	14.4/21 (69)	Articles published in STARD-adopting journals had a significantly higher total STARD score (15.4 vs. 14.1; *p* = 0.018 than STARD-nonadopting journals. From 2003 to 2011, the total STARD score increased by an average of 0.30 points (*p* = 0.03) per year

Reports are listed according to appearance in text

*STARD* Standards for Reporting Diagnostic Accuracy; *IF* impact factor; *OR* odds ratio; *CI* confidence interval

*Indicates the use of the STARD 2015 checklist and guidelines

^a^Unless otherwise indicated, numbers are STARD items reported and data in parentheses are percentages

^b^No absolute numbers for mean total STARD score mentioned in text

^c^Results from 2000 to 2004 are from Smidt et al. [30]

Our study has some potential limitations. First, we searched MEDLINE using a validated search strategy to identify relevant diagnostic accuracy studies. Since the search strategy has 80.0% sensitivity and 97.3% specificity [18], some studies may not have been recognized by our search filter. We minimized this risk by additionally identifying further studies by a manual search of the website. Second, we excluded item 11 with the qualifier “if alternatives exist” from the original STARD 2015 checklist for reasons mentioned above. This may have affected the results of our analysis depending on the performance of item 11. Additionally, we focused on a single journal to be able to draw direct comparisons after a policy change in 2016. Due to these two points, the generalizability of our results may be limited and further studies in journals making such policy change are warranted. Also, by choosing articles published in 2019 instead of 2020 or 2021, the immediacy of our data might be affected. We made this decision due to the ongoing COVID-19 crisis since 2020, which brought a great increase in submissions about this single topic with reductions in diagnostic accuracy studies. Third, we were rather strict in assigning scores. For example, baseline characteristics (item 20) were only judged as being satisfactorily reported when some information other than sex and age, such as underlying conditions, was also provided. In addition, several items are prone to subjective assessment. To reduce rater bias, we explicitly defined each item, did pilot exercises, and resolved discrepancies in consensus meetings. Finally, the update of STARD was released in October 2015. Consequently, some authors of studies published in 2015 may not yet have had access to the revised checklist. Nevertheless, we decided to use this list for all studies because the update was intended to facilitate the use of STARD and to highlight items prone to bias and variation, as suggested by recent evidence [8]. Interestingly, five of nine new checklist items were already frequently reported in our study sample: Item 2 (structured summary), 3 (clinical background), 26 (study limitations), 27 (implications for practice), and 30 (sources of funding), which may suggest that reporting these items has already been adopted.

In conclusion, our results showed overall adherence to reporting guidelines in diagnostic accuracy studies to be moderate to good. With the STARD guidelines being mandatory since 2016, studies published in 2019 had a relevantly higher total STARD score than those published in 2015. Making the STARD guidelines mandatory may thus positively affect the reporting quality of diagnostic accuracy studies. This should encourage journals and publishers to add mandatory reporting guidelines to their author instructions.

## Supplementary Information

**Additional file 1**: Full search strategy and list of STARD scores for all included articles.

## Data Availability

Used data sets analyzed during the study are available from the corresponding author upon reasonable request.

## Abbreviations

CI: Confidence interval
IQR: Interquartile range
STARD: Standards for Reporting Diagnostic Accuracy
