# Supplementary material for: Has the quality of reporting improved since it became mandatory to use the Standards for Reporting Diagnostic Accuracy?
Source: Insights Imaging. 2023 May 15;14:85. doi: 10.1186/s13244-023-01432-7 (PMC10184623; doi:10.1186/s13244-023-01432-7)
Supplement: Supplementary file 1 — Additional file 1: Full search strategy and list of STARD scores for all included articles. [file 13244_2023_1432_MOESM1_ESM.pdf]

Has the quality of reporting improved since it became mandatory to use the Standards for Reporting Diagnostic Accuracy?

Table S1. Search filter of Devillé et al. for publications of diagnostic accuracy studies in MEDLINE [18].

Search Filter	Content	Sensitivity % (*n*=75)	Specificity % (*n*=2392)
Haynes' sensitive strategy	sensitivity and specificity (exploded) (sh)<br>diagnosis& (sh)<br>diagnostic use (sh)<br>sensitivity (tw)<br>specificity (tw)	73.3 (63.3–83.3)	94.3 (93.3–95.2)
Strategy 3<br>(basis for our search strategy)	sensitivity and specificity (exploded) (sh)<br>specificity (tw)<br>false negative (tw)<br>accuracy (tw)	80.0 (71.0–89.1)	97.3 (96.6–97.9)

Unless otherwise indicated, data in parentheses are 95% confidence intervals.
sh, Medline subheading; tw, text word [18].

Table S2. Full search strategy in MEDLINE using PubMed for diagnostic accuracy studies published in Radiology in 2015 and 2019. Search strategy is based on findings of Devillé et al. [18].

1	sensitivity and specificity (MeSH)
3	specificity.tw.
4	false negative.tw.
5	accuracy.tw.
6	1 or 2 or 3 or 4 or 5
7	Radiology jn.
8	6 and 7
9a	2015/01/01-2015/12/31 (Date-Publication)
9b	2019/01/01-2019/12/31 (Date-Publication)
10a	8 and 9a
10b	8 and 9b

MeSH, Medical Subject Heading; tw., text word; jn., journal.

Table S3. Complete list of all 66 diagnostic accuracy studies in our analysis with individual total STARD scores.

Publication Year	Total STARD Score*	First Author	Journal	DOI
2015	22.5	Yang	Radiology	10.1148/radiol.2015141126
2015	22	Habis	Radiology	10.1148/radiol.14140861
2015	21	Hwang	Radiology	10.1148/radiol.15141350
2015	21	Park J.J.	Radiology	10.1148/radiol.14140920
2015	20.5	Lee D.H.	Radiology	10.1148/radiol.14140141
2015	20	Zheng	Radiology	10.1148/radiol.14140828
2015	19.5	Correas	Radiology	10.1148/radiol.14140567
2015	19.5	Elkrief	Radiology	10.1148/radiol.14141210
2015	19.5	Harris	Radiology	10.1148/radiol.14140763
2015	19.5	Mileto	Radiology	10.1148/radiol.14140876
2015	19.5	Park J.E.	Radiology	10.1148/radiol.14141414
2015	19	Ba-Ssalamah	Radiology	10.1148/radiol.2015142366
2015	18.5	Atri	Radiology	10.1148/radiol.2015140907
2015	18.5	Li	Radiology	10.1148/radiol.14140911
2015	18.5	Mailhot	Radiology	10.1148/radiol.2015142413
2015	18.5	Schipper	Radiology	10.1148/radiol.14141167
2015	18.5	Tang	Radiology	10.1148/radiol.14140754
2015	18	Coolen	Radiology	10.1148/radiol.14132111
2015	18	Luo	Radiology	10.1148/radiol.2015141856
2015	18	Samir	Radiology	10.1148/radiol.14140839
2015	18	Suh	Radiology	10.1148/radiol.15141216
2015	18	Wallihan	Radiology	10.1148/radiol.14140468
2015	17.5	Cochet	Radiology	10.1148/radiol.14141140
2015	17.5	Ohno	Radiology	10.1148/radiol.14132289
2015	17.5	Pasoglou	Radiology	10.1148/radiol.14141242
2015	17	Kim S.H.	Radiology	10.1148/radiol.14132960
2015	16.5	Zhou	Radiology	10.1148/radiol.2015142309
2015	16	Kim H.J.	Radiology	10.1148/radiol.14140390

2015	16	Lee J.R.	Radiology	10.1148/radiol.14141073
2015	15	Holzapfel	Radiology	10.1148/radiol.14140510
2015	15	Priola	Radiology	10.1148/radiol.14132665
2015	15	Strobel	Radiology	10.1148/radiol.14140645
2015	14.5	Darnell	Radiology	10.1148/radiol.15141132
2015	14.5	Liu	Radiology	10.1148/radiol.14140481
2015	14.5	Samir	Radiology	10.1148/radiol.2015141627
2015	14.5	Sun	Radiology	10.1148/radiol.15141625
2015	14.5	Zhao	Radiology	10.1148/radiol.14132687
2015	14	Bogner	Radiology	10.1148/radiol.14132340
2015	13	Barr	Radiology	10.1148/radiol.14132404
2019	24.5	Grob	Radiology	10.1148/radiol.2019182666
2019	22.5	Baessler	Radiology	10.1148/radiol.2019190101
2019	22.5	Kim Y.Y.	Radiology	10.1148/radiol.2019181995
2019	22.5	Kronlage	Radiology	10.1148/radiol.2019182538
2019	22	Son	Radiology	10.1148/radiol.2019190035
2019	21.5	Meyer	Radiology	10.1148/radiol.2019181100
2019	21.5	Tavare	Radiology	10.1148/radiol.2018180950
2019	21.5	Wildman-Tobriner	Radiology	10.1148/radiol.2019182128
2019	21	Lee	Radiology	10.1148/radiol.2019182867
2019	20	Booz	Radiology	10.1148/radiol.2018181286
2019	20	Chassagnon	Radiology	10.1148/radiol.2019182099
2019	20	Wu	Radiology	10.1148/radiol.2018181168
2019	19.5	Fontaine	Radiology	10.1148/radiol.2019182457
2019	19.5	Schelb	Radiology	10.1148/radiol.2019190938
2019	19.5	Wang	Radiology	10.1148/radiol.2019182506
2019	19	Hwang	Radiology	10.1148/radiol.2019191225
2019	19	Lennartz	Radiology	10.1148/radiol.2018181567
2019	19	Zhou	Radiology	10.1148/radiol.2019181729
2019	18.5	Bahl	Radiology	10.1148/radiol.2019181637
2019	18.5	Muehlematter	Radiology	10.1148/radiol.2019190687
2019	18.5	Sumkin	Radiology	10.1148/radiol.2019190887

2019	18.5	Sung	Radiology	10.1148/radiol.2019182660
2019	18	Jensen	Radiology	10.1148/radiol.2018181657
2019	18	Yang	Radiology	10.1148/radiol.2019181816
2019	17.5	Kim D.H.	Radiology	10.1148/radiol.2019182587
2019	17.5	Mushtaq	Radiology	10.1148/radiol.2019181959
2019	17.5	Yeh	Radiology	10.1148/radiol.2019182122

STARD, Standards for Reporting Diagnostic Accuracy; DOI, Digital Object Identifier.
* Maximum of 29 points.
